# Targeted Next-Generation Sequencing for Improved Clinical Outcomes in People Living With Rare Diseases in Global South: Protocol for a Systematic Review and Meta-Synthesis

**DOI:** 10.2196/85150

**Published:** 2026-07-03

**Authors:** Mapaseka Seheri, Dini Mawela, Lerato Kgosana, Chantelle Baker, Wesley Van Hougenhouck-Tulleken, Olanrewaju Oladimeji

**Affiliations:** 1 Diarrheal Pathogens Research Unit (DPRU), Department of Medical Virology, School of Medicine Sefako Makgatho Health Sciences University Pretoria, Gauteng South Africa; 2 Department of Paediatrics and Child Health, School of Medicine Sefako Makgatho Health Sciences University Pretoria, Gauteng South Africa; 3 Electron Microscope Unit, School of Medicine Sefako Makgatho Health Sciences University Pretoria, Gauteng South Africa; 4 Department of Nephrology, School of Medicine Sefako Makgatho Health Sciences University Pretoria, Gauteng South Africa; 5 Department of Public Health Sefako Makgatho Health Sciences University Pretoria, Gauteng South Africa; 6 SAMRC/SMU Public Health Interventions, Innovations and lmplementation Research Unit Sefako Makgatho Health Sciences University Pretoria, Gauteng South Africa

**Keywords:** Global South, targeted next-generation sequencing, rare diseases, diagnostic yield, genomic medicine

## Abstract

**Background:**

Rare diseases affect many individuals and pose major challenges in diagnosis and treatment, especially in Global South countries where health care resources are limited. Targeted next-generation sequencing (NGS) has significantly advanced diagnostic accuracy and clinical care for rare diseases globally; however, its implementation and impact within the Global South context remain insufficiently studied.

**Objective:**

This study aims to evaluate the use, clinical benefits, challenges, and implementation outcomes of targeted NGS for diagnosing and managing rare diseases in Global South populations. Specifically, it seeks to quantify the diagnostic yield of NGS, examine its influence on subsequent clinical decision-making, and identify principal barriers to, and facilitators of, the implementation of targeted NGS approaches in these contexts.

**Methods:**

This protocol follows the PRISMA (Preferred Reporting Items for Systematic Reviews and Meta-Analyses) guidelines. We will systematically search PubMed, Scopus, and Web of Science for studies published between 2005 and 2025 that report on the use of targeted NGS in Global South population with rare diseases. Two reviewers will independently perform study selection, data extraction, quality assessment, and evaluation of risk of bias by using QUADAS-2 for diagnostic accuracy studies and the risk of bias assessment tool for nonrandomized studies. Meta-analyses will be conducted to estimate pooled outcomes for diagnostic yield, with heterogeneity assessed using random effects models. Heterogeneity will be further examined through visual inspection of forest plots and by evaluating the chi-square test and *I*² statistic.

**Results:**

The protocol has been registered with PROSPERO (CRD420251078455). Database search or screening, data extraction, and data synthesis are planned to commence in June 2026 and conclude by September 2026. Study findings will synthesize the diagnostic yield, clinical impact, and contextual determinants influencing the implementation of targeted NGS in Global South health care settings.

**Conclusions:**

This review will provide evidence on the application, advantages, limitations, and clinical outcomes of targeted NGS for individuals affected by rare diseases in countries of the Global South. The finding will identify priorities for capacity strengthening, policy development, and future genomic research.

**Trial Registration:**

PROSPERO CRD420251078455; https://www.crd.york.ac.uk/PROSPERO/view/CRD420251078455

**International Registered Report Identifier (IRRID):**

PRR1-10.2196/85150

## Introduction

### Background

Rare diseases, although individually uncommon, collectively affect millions of people worldwide, often leading to morbidity and early mortality [1]. These diseases are typically defined as conditions that affect a small percentage of the population, fewer than 1 in 2000 individuals [2]. Rare diseases present unique challenges in diagnosis and management, especially in low- and middle-income countries (LMICs) such as those in Global South [3]. In Global South countries, the burden of rare diseases is worsened by limited awareness, underresourced health care systems, and substantial barriers to timely and accurate diagnosis [4]. In this protocol, Global South refers to LMICs, including countries in sub-Saharan Africa, Latin America, parts of Asia, and the Middle East.

Rare diseases are described as a highly diverse group of disorders that can affect any body system. Most of these diseases are genetic disorders, often severely disabling, shortening life expectancy, and impairing physical and mental abilities, resulting in disabilities that reduces quality of life and limit educational and earning potential [5]. Rare diseases constitute a significant public health concern and pose substantial challenges to the medical community. They are often referred to as “health orphans,” reflecting their historical marginalization and the longstanding neglect they have received in research [6].

Traditional diagnostic approaches, which often involve a lengthy and costly diagnostic journey, are insufficient for rare diseases, resulting in delays in treatment and substantial emotional and financial strain for affected individuals and families [7]. In this context, targeted next-generation sequencing (NGS) has emerged as a powerful tool for enhancing the diagnostic return and clinical management of rare diseases [8]. For instance, at Tygerberg Hospital in South Africa, the application of NGS gene panels achieved a diagnostic yield of 39.5% for adult-onset neurogenetic disorders across 22 distinct disease categories [9], allowing targeted testing for relatives, improved management, and genetic counseling in the Global South context. Unlike conventional methods, targeted NGS panels focus on sequencing a select set of genes known to be associated with specific disease phenotypes, making the approach faster, more cost-effective, and highly sensitive [10]. While whole exome and whole genome sequencing are becoming increasingly available, they remain an inferior option in resource-limited settings due to high-related costs. The application of targeted NGS in clinical settings enables early and precise diagnosis, which is critical for guiding appropriate medical management, offering prognostic insights, facilitating genetic counseling, and potentially identifying candidates for emerging therapies [11]. Furthermore, in populations characterized by high genetic diversity, such as those across Global South, integrating targeted NGS into clinical practice could illuminate novel genetic variants and expand the global understanding of rare diseases [12].

Despite its potential, access to targeted NGS remains limited across the Global South countries due to infrastructural, financial, and technical challenges [13]. Capacity building through collaborative efforts, increased investment in genomics research, and the development of locally relevant gene panels are crucial steps toward making precision medicine a reality for Africans living with rare diseases [14]. Moreover, initiatives such as Human Heredity and Health in Africa (H3Africa) have demonstrated that with appropriate investment and training, African researchers and clinicians can successfully implement genomic technologies to address health disparities [15].

The objective of this study is to systematically review and synthesize evidence on the use, benefits, challenges, and clinical outcomes associated with targeted NGS for people living with rare diseases in Global South. This will highlight the current gaps in rare disease diagnosis on the continent, examine the benefits and challenges associated with deploying targeted NGS, and discuss strategies for integrating this technology into Global South health care systems to ensure more equitable health outcomes. The Population, Intervention, Comparison, and Outcome framework will be used during the literature search: populations: individuals with rare diseases in Global South settings, health workers, and policy makers; intervention: targeted NGS panels; comparator: usual diagnostic approaches or alternative genetic testing; and outcomes: diagnostic yield, clinical management changes, and implementation barriers or facilitators.

### Research Questions

This systematic review is designed to investigate the following research questions: (1) What is the diagnostic yield of targeted NGS for rare diseases in Global South populations? (2) How does targeted NGS influence clinical management, including treatment decisions and genetic counseling? (3) What barriers and facilitators affect the implementation of targeted NGS in Global South health care systems?

## Methods

### Overall Approach

A systematic review will be conducted in accordance with the PRISMA (Preferred Reporting Items for Systematic Reviews and Meta-Analyses) guidelines [16], focusing on studies that explore the application, benefits, challenges, and clinical outcomes of targeted NGS for individuals living with rare diseases in Global South. This process will detail the methods used for study identification, selection, and inclusion. Any amendments affecting eligibility criteria, outcomes, data extraction, or analysis made to this protocol during the review will be documented, along with the date of each amendment, with justification recorded in the PROSPERO audit trail and a version-controlled log. All amendments will be reported in the final review. The protocol is reported according to the PRISMA-P (PRISMA-Protocols) (Multimedia Appendix 1).

### Population of Interest

The population of interest includes individuals of all ages living with rare diseases across Global South countries. This comprises patients who have undergone or are candidates for targeted NGS as part of their diagnostic assessment or clinical management. This study will focus on multigene sequencing panels and targeted capture-based approaches to investigate specific disease-associated genes. Studies focused on single-gene Sanger sequencing will not be included in the review. Studies involving Global South populations diagnosed with rare genetic disorders, or where targeted NGS was used to investigate suspected rare diseases within clinical or research settings, will be included. Both pediatric and adult populations will be considered, regardless of gender, ethnicity, or socioeconomic status.

### Search Strategy

Comprehensive search will be conducted across multiple electronic databases to identify relevant studies. Databases to be searched include PubMed, Scopus, Web of Science, Embase, and African Journals Online. Google Scholar will also be searched to increase the chances of finding more relevant articles related to application, benefits, challenges, and clinical outcomes of targeted NGS for individuals living with rare diseases in Global South. Additional sources such as gray literature, conference abstracts, and reference lists of included studies will also be screened to capture any studies not indexed in major databases. To successfully retrieve relevant articles, the search string shown in Table 1 were used during the pilot search from 3 databases.

**Table 1:** Table of pilot search from PubMed, Scopus, and Web of Science (N=767).

Database	Search string	Number of articles found
PubMed	“Targeted NGS OR TNGS OR targeted resequencing OR targeted enrichment sequencing OR targeted gene panel sequencing OR sub-panel NGS OR gene specific NGS) AND (Clinical Outcomes OR clinical results OR health outcomes OR clinical findings OR patient outcomes)) AND (Rare diseases OR uncommon diseases OR rare disorders OR rare illness OR special conditions OR orphan diseases)) AND (Global south OR sub-Sahara Africa OR Africa OR Latin America OR Asia OR Oceania, ) AND (2005/1/1:2025/12/31)”	687
Web of Science	“Targeted next generation sequencing OR TNGS OR targeted resequencing OR targeted enrichment sequencing OR targeted gene panel sequencing OR sub-panel NGS OR gene specific NGS (Topic) AND Clinical Outcomes OR clinical results OR health outcomes OR clinical findings OR patient outcomes (All Fields) AND Rare diseases OR uncommon diseases OR rare disorders OR rare illness OR special conditions OR orphan diseases (All Fields) AND Global south OR sub-Sahara Africa OR Africa OR Latin America OR Asia OR Oceania”	54
Scopus	“TITLE-ABS-KEY ( Targeted next generation sequencing ) OR TITLE-ABS-KEY ( TNGS ) OR TITLE-ABS-KEY ( targeted resequencing ) OR TITLE-ABS-KEY ( targeted enrichment sequencing ) OR TITLE-ABS-KEY ( targeted gene panel sequencing ) OR TITLE-ABS-KEY ( sub-panel NGS ) OR TITLE-ABS-KEY ( gene specific NGS ) AND TITLE-ABS-KEY ( Clinical Outcomes ) OR TITLE-ABS-KEY ( clinical results ) OR TITLE-ABS-KEY ( health outcomes ) OR TITLE-ABS-KEY ( clinical findings ) OR TITLE-ABS-KEY ( patient outcomes ) AND TITLE-ABS-KEY ( Rare diseases ) OR TITLE-ABS-KEY ( uncommon diseases ) OR TITLE-ABS-KEY ( rare disorders ) OR TITLE-ABS-KEY ( rare illness ) OR TITLE-ABS-KEY ( special conditions ) OR TITLE-ABS-KEY ( orphan diseases ) AND TITLE-ABS-KEY ( Global south ) OR TITLE-ABS-KEY ( sub-Sahara Africa ) OR TITLE-ABS-KEY ( Africa) OR TITLE-ABS-KEY (Latin America) OR TITLE-ABS-KEY (Asia) OR TITLE-ABS-KEY (Oceania)”	26

### Article Screening

**Figure 1:**
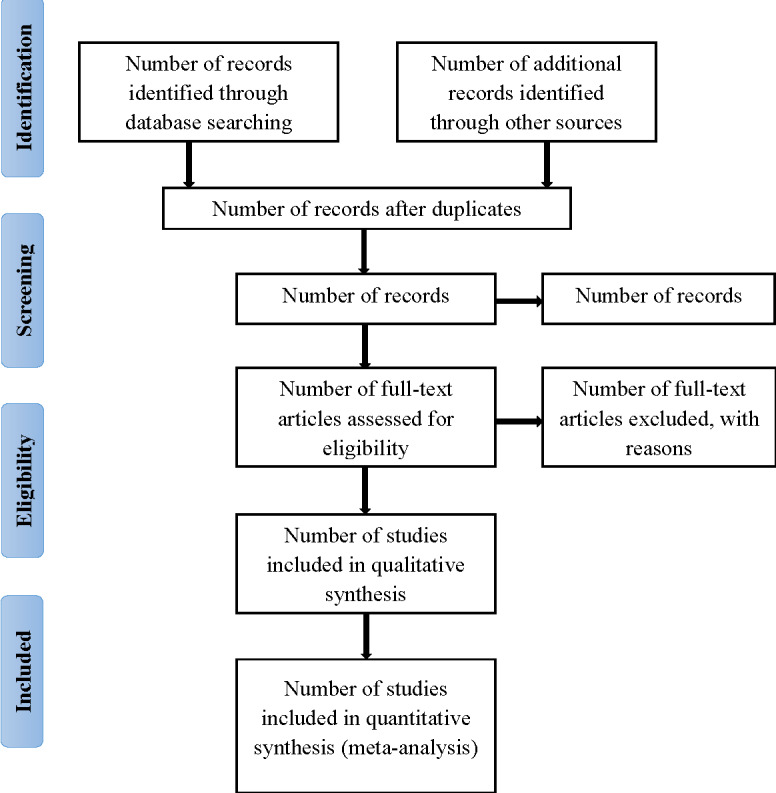
PRISMA (Preferred Reporting Items for Systematic Reviews and Meta-Analyses) flowchart of the systematic search process.

The screening of articles for this systematic review and meta-analysis will be conducted in 2 distinct stages: title and abstract screening, followed by full-text screening. After completing the database search, all retrieved records will be imported into a reference management software program, such as EndNote or Mendeley, where duplicate records will be identified and removed. The screening process will be implemented using specialized systematic review management platforms, such as Rayyan or Covidence, with comprehensive audit trails documented and preserved throughout all stages of the screening. Title and abstract screening will then be performed independently by 2 reviewers, who will assess each record for its relevance according to the predefined eligibility criteria. Articles will be classified as either “include,” “exclude,” or “uncertain” based on the information available in the title and abstract. Studies that clearly do not meet the inclusion criteria will be excluded, while those that appear relevant or for which relevance is unclear will proceed to full-text screening. Any discrepancies between the 2 reviewers during this stage will be resolved through discussion, and if necessary, consultation with a third reviewer will be sought to achieve consensus. During the full-text screening phase, the same 2 reviewers will independently assess the full articles of all studies classified as “include” or “uncertain” during the initial screening. Each article will be reviewed in detail to determine its eligibility for inclusion in the final systematic review. If an article is excluded at this stage, the reason for its exclusion will be recorded in a standardized form to ensure transparency. Common reasons for exclusion may include the study not focusing on targeted NGS, not being conducted within Global South population, or failing to report clinical outcomes related to rare diseases. The screening process will be systematically documented using the PRISMA 2020 flow diagram in Figure 1, which will visually depict the number of records identified, screened, excluded, and included at each stage, along with justifications for any exclusions at the full-text review level. This rigorous approach will ensure that the article selection process is transparent, reproducible, and in line with best practices for conducting systematic reviews.

### Primary Outcome and Determinants

The primary outcome of this systematic review and meta-analysis is evidence of clinical utility of targeted NGS among people living with rare diseases in Global South, with diagnostic yield as the main quantitative measure. Clinical outcomes will include, but are not limited to, the accuracy and timeliness of diagnosis, the initiation of appropriate medical management based on genetic findings, prognostic information provided to patients and families, and access to relevant therapeutic interventions. Studies that report on the clinical utility of targeted NGS, such as its impact on diagnosis rates, changes in patient management, or contributions to family planning and genetic counseling, will be prioritized in the analysis. Determinants that will be considered include various factors influencing the successful implementation and clinical effectiveness of targeted NGS. These may include the availability and accessibility of genomic technologies, the quality of health care infrastructure, the presence of trained personnel in genetic diagnostics, and the socioeconomic and policy environments supporting genomics research and clinical application in Global South countries. Additional determinants will include barriers such as cost, technical capacity, cultural perceptions of genetic testing, and ethical considerations surrounding data use and patient privacy. The review will also explore enablers, such as regional and international collaborations, governmental support, and initiatives such as H3Africa, which aim to promote genomics research and precision medicine on the continent. Through a detailed synthesis of the primary outcomes and determinants, the systematic review seeks to provide a comprehensive understanding of how targeted NGS can influence the care and prognosis of individuals with rare diseases in Global South, while identifying gaps and opportunities for strengthening clinical implementation strategies.

### Inclusion and Exclusion Criteria

Studies will be included if they focus on the use of targeted NGS to diagnose or manage rare diseases among populations in Global South countries. Rare disease is defined as affecting fewer than 1 in 2000 individuals, while targeted NGS refers to sequencing of predefined gene panels and Global South is defined as LMICs based on the World Bank classification. Eligible studies must report clinical outcomes, such as diagnostic yield, changes in patient management, or access to therapy resulting from targeted NGS applications. Both observational studies (including cohort, cross-sectional, and case series) and interventional studies will be considered for inclusion. Furthermore, studies published in English, conducted from the year 2005 onward, and involving human participants will be eligible. Studies that address barriers, facilitators, or implementation strategies for the use of targeted NGS in Global South health care systems will also be included.

Studies will be excluded if they do not involve Global South population or if they focus solely on whole genome sequencing or whole exome sequencing without specific analysis of targeted NGS panels. Articles that are purely technical in nature, such as those describing sequencing methods without any clinical application, will be excluded. Conference abstracts without full-text availability, editorials, commentaries, reviews, and opinion pieces will also not be considered. In addition, studies published in languages other than English, and those with insufficient data on clinical outcomes or implementation context, will be excluded from the review.

### Data Extraction

A structured data extraction process will be used to ensure consistency and accuracy across studies. Data from all eligible articles will be independently extracted by 2 reviewers using a standardized extraction form developed for this review. The extracted information will include the study title, first author, year of publication, country or countries where the study was conducted, study design, study population characteristics, sample size, type of rare diseases investigated, and the specific targeted NGS panels or technologies used. Information on diagnostic yield, time to diagnosis, clinical impact on patient management, and access to therapeutic interventions resulting from the use of targeted NGS will also be collected. Diagnostic yield is defined as the proportion of individuals receiving a confirmed molecular diagnosis. In addition, the reviewers will extract details about reported barriers and facilitators to the use of targeted NGS, implementation strategies, and funding sources where applicable. Any discrepancies between the 2 reviewers will be resolved through discussion or by consulting a third reviewer if necessary. In instances where important data are missing or unclear, attempts will be made to contact the study authors for clarification. The extracted data will be compiled into a central database to allow for systematic analysis and synthesis.

Quantitative outcomes will be reported descriptively, and pooled meta-analysis will be used where appropriate. For outcomes that are not suitable for pooling, synthesis will follow the synthesis without meta-analysis principle [17]. Qualitative outcomes such as barriers, facilitators, acceptability, feasibility, resource constraints, and workforce capacity will follow a structured narrative synthesis following structured narrative synthesis informed by established methodological frameworks. In addition, a thematic synthesis comprising systematic coding of extracted text, generation of descriptive themes, and development of higher-order analytics themes will be followed [18].

### Risk of Bias Assessment and Quality Assessment

Risk of bias will be assessed qualitatively, focusing on critical domains including selection bias related to the sample population, selection bias concerning the participation rate, reporting bias from selective outcome reporting, performance bias in terms of analytical methods for bias control, and other potential sources of bias. Each study will be rated as having a low, unclear, or high risk of bias using the risk of bias assessment tool for nonrandomized studies, such as ROBIS [19]. This structured approach will enable a transparent evaluation of the internal validity of each included study. In parallel with the risk of bias assessment, the quality of the included studies will be appraised using the JBI checklist for studies reporting prevalence data [20]. Where possible, diagnostic accuracy studies will additionally be assessed using the QUADAS-2 tool. The evaluation will consider whether the sample frame was appropriate to address the target population, whether participants were sampled appropriately, whether the sample size was sufficient, and whether the study participants and setting were clearly described. It will also assess whether the data analysis was conducted with adequate coverage of the identified sample, whether valid methods were used to identify the condition of interest, whether the condition was measured in a standard and reliable way across all participants, whether statistical analyses were appropriate, and whether the response rate was adequate and handled appropriately if it was low. For each of these criteria, responses will be classified as “yes,” “no,” “unclear,” or “not applicable.” This thorough process will ensure that the final body of evidence included in the review is both methodologically sound and relevant to informing clinical outcomes for people living with rare diseases in Global South.

### Statistical Analysis

The statistical analysis for this systematic review will present the extracted data in a descriptive table, which will include key study characteristics. These characteristics will include the authors, year of the study, location, study participants, article type, gender stratification, level of education, sample size, number of participants, mean age, and the prevalence of outcomes. The pooled prevalence of the use of targeted NGS and its associated clinical outcomes for individuals living with rare diseases in Global South will be calculated using STATA (version 17; StataCorp) [21]. Heterogeneity across studies will be evaluated using the *I*² statistic, following the DerSimonian-Laid random effects model. The *I*² values will be interpreted to assess the degree of variation in the results that is attributable to real differences between studies, as opposed to random chance. A low *I*² value indicates minimal heterogeneity, while moderate and high values suggest increasing variability across studies. Specifically, *I*² values of ≤25% will be considered low, around 50% as moderate, and values >75% as high. Where data permit, studies will be grouped according to variables, such as the general population, male, and female, to explore any subgroup variations in prevalence. The prevalence estimates will be accompanied by 95% CIs to provide a measure of the precision of the estimates. A *P* value of <.05 will be considered to indicate significant heterogeneity among the studies. Sensitivity analyses will be carried out by excluding individual studies in a stepwise manner to evaluate the impact of each study on the overall analysis. This will help determine whether any specific study is influencing the findings substantially. To assess potential publication bias, funnel plots will be visually inspected for asymmetry, and the Egger linear regression test will be applied [22]. Finally, meta-regression will be performed to investigate how factors such as the year of study and sample size might affect the observed prevalence of targeted NGS use and its clinical outcomes in people with rare diseases in Global South. Meta-analysis will only be conducted when there is sufficient homogeneity in the study design, outcome, and reporting. Alternatively, where pooling is not possible, the finding will be synthesized narratively.

### Ethical Considerations and Dissemination

The protocol has obtained waiver from the ethics committee of Sefako Makgatho Health Sciences University (SMUREC/M/364/2025). The ethical considerations for this systematic review will adhere to the highest standards of research integrity and respect for individuals involved in the studies being reviewed. As this review focuses on secondary data analysis, ethics approval is not required for the review itself. However, all the primary studies included in the review must have received appropriate ethics approval from their respective ethics committees or institutional review boards.

The findings of this review will be disseminated to a wide audience to maximize their impact. The results will be shared through peer-reviewed journals, conferences, and workshops focusing on rare diseases, genomics, and health care in Global South. Additionally, we will engage with stakeholders, such as health care practitioners, policymakers, and public health experts in Global South, to ensure that the findings are translated into practical recommendations for improving clinical outcomes for individuals with rare diseases. The review’s results will also be disseminated to research institutions and organizations working on rare diseases in LMICs, particularly those involved in genomic medicine and precision health care. Finally, efforts will be made to share the results with the broader scientific community and the public through open-access platforms, ensuring the broadest possible reach and impact of the research findings.

## Results

This review protocol has been prospectively registered with PROSPERO (registration ID: CRD420251078455). Systematic database searching, study selection, and data extraction procedures will commence following publication of this protocol. Narrative synthesis will form the primary analytical approach, with quantitative meta-analysis performed only where sufficient methodological comparability and outcome homogeneity are identified. Literature searches are expected to commence between May and June 2026, while title and abstract screening will be performed between June and July. Full-text screening and data extraction as well as risk of bias analysis are planned for July to August 2026 and September to October 2026, respectively. Evidence synthesis and manuscript drafting will be undertaken between October and November 2026.

## Discussion

### Principal Findings

The systematic review and meta-analysis study examining the application of targeted NGS for improving clinical outcomes in people living with rare diseases in Global South seeks to explore how genomic technologies can enhance the diagnosis and management of these conditions on the continent. This discussion provides insights into the significance of the study, the challenges faced by people with rare diseases, and the potential impact of targeted NGS in overcoming these obstacles. Rare diseases are a significant public health issue globally, and their burden in Global South is particularly profound [1,7]. The review will highlight the unique challenges posed by rare diseases in Global South countries, where limited awareness, underresourced health care systems, and a lack of access to advanced diagnostic tools make it difficult to provide timely and accurate diagnoses [3,4]. This often results in delayed or inappropriate treatment, contributing to prolonged suffering, unnecessary health care costs, and poor clinical outcomes [7] This technology offers a promising solution to these challenges by providing more accurate, faster, and cost-effective diagnostic alternatives compared to traditional methods [8,11].

The study will specifically focus on the clinical outcomes associated with the use of targeted NGS in Global South populations. By examining data on diagnostic accuracy, treatment response, and patient outcomes, the study aims to assess whether the application of NGS leads to improved clinical management of rare diseases [11]. This approach is particularly important in the Global South context, where many rare diseases remain poorly understood due to a lack of localized genetic research and the underrepresentation of Global South population in global genomic databases [12]. By identifying genetic variants unique to Global South population, targeted NGS can contribute to a better understanding of these conditions and inform the development of tailored treatments and therapies. Another key aspect of this study will be the emphasis on understanding the barriers to the implementation of targeted NGS in Global South health care systems. Despite the promising potential of this technology, access remains limited due to infrastructural, financial, and technical challenges [13,14]. These barriers include the high cost of NGS technologies, limited availability of trained personnel, and the need for specialized laboratory infrastructure.

This study will acknowledge the role of collaborative efforts in overcoming these obstacles. Initiatives such as H3Africa have demonstrated the feasibility of implementing genomic research and genomic medicine in Africa, showing that with appropriate investment and training, African researchers and health care providers can successfully use genomic technologies [15]. The study will explore how such collaborations can be expanded to integrate targeted NGS into routine clinical practice, helping to bridge the gap in rare disease diagnosis and care.

In terms of methodology, the systematic review and meta-analysis will provide a robust synthesis of the available evidence, focusing on studies that assess the use, benefits, and challenges of targeted NGS in Global South population. By pooling data from multiple studies, the review aims to offer a comprehensive understanding of the clinical outcomes associated with this technology. The use of rigorous inclusion and exclusion criteria, along with the application of quality assessment tools, will ensure that the results are reliable and applicable to the Global South context. The findings of this review will have the potential to inform health care strategies, genomic research initiatives, and capacity-building efforts aimed at reducing the health disparities faced by the Global South population living with rare diseases.

### Strengths and Limitations of This Study

The strengths of this systematic review and meta-analysis study lie in its comprehensive approach to evaluating the use of targeted NGS in improving clinical outcomes for individuals with rare diseases in Global South. By synthesizing data from diverse studies, the review aims to provide a broad and reliable understanding of how targeted NGS can enhance diagnostic accuracy, treatment response, and patient outcomes. The focus on clinical outcomes, such as diagnostic accuracy and treatment responses, makes the study highly relevant for health care providers and policymakers. It will also offer insights into the genetic variants unique to the Global South population, potentially leading to more tailored and effective treatments. The review’s inclusion of a wide range of studies from different Global South countries ensures that it reflects the specific challenges and opportunities in the Global South context, including the underrepresentation of Global South populations in global genomic databases. In addition, the study will focus on identifying barriers to implementing targeted NGS in Global South, such as infrastructural, financial, and technical challenges, and its emphasis on collaborative initiatives such as H3Africa that have successfully integrated genomic research and medicine into clinical practice.

However, the study has limitations, including the potential scarcity of studies focusing on Global South population, which may impact the generalizability of the findings. The heterogeneity of the included studies could introduce variability in the results, making definitive conclusions challenging. Moreover, reliance on published studies may introduce publication bias, and the rapidly evolving nature of genomic technologies may render the findings outdated if newer studies are not included, and the review will primarily focus on targeted NGS, potentially overlooking other genomic technologies that could benefit rare disease diagnosis and treatment. Despite these limitations, the study will provide a robust framework for evaluating targeted NGS’s potential to improve clinical outcomes for rare diseases in Global South and will have the potential to contribute valuable evidence to the growing field of genomic medicine on the continent.

## Data Availability

No extracted dataset or analytic code is available at this stage. After review and publication, technical appendix, statistical code, and dataset will be made available from the Dryad repository.

## Appendix

Multimedia Appendix 1 PRISMA-P 2015 Checklist. This appendix presents the Preferred Reporting Items for Systematic Review and Meta-Analysis Protocols (PRISMA-P) 2015 checklist for this study.

## Abbreviations

H3Africa: Human Heredity and Health in Africa
LMIC: low- and middle-income country
NGS: next-generation sequencing
PRISMA: Preferred Reporting Items for Systematic Reviews and Meta-Analyses
PRISMA-P: Preferred Reporting Items for Systematic Reviews and Meta-Analyses Protocols
