# Supplementary material for: Targeted Next-Generation Sequencing for Improved Clinical Outcomes in People Living With Rare Diseases in Global South: Protocol for a Systematic Review and Meta-Synthesis
Source: JMIR Res Protoc. 2026 Jul 3;15:e85150. doi: 10.2196/85150 (PMC13379697; doi:10.2196/85150)
Supplement: Multimedia Appendix 1 [file resprot_v15i1e85150_app1.docx]

Appendix 2. PRISMA-P 2015 Checklist. This appendix presents the Preferred Reporting Items for Systematic Review and Meta-Analysis Protocols (PRISMA-P) 2015 checklist for this study.

| **Section / Topic** | **Item No.** | **Checklist item** | **Reported** | **Page number(s)** |
| --- | --- | --- | --- | --- |
| Title | 1a | Identify the report as a protocol of a systematic review | Yes | 1 |
| Title | 1b | If the protocol is for an update of a previous review, identify as such | No | N/A |
| Registration | 2 | Provide registration information, including registry name and registration number | Yes | 2, 14 |
| Authors | 3a | Provide name, institutional affiliation, and email address of all protocol authors | Yes | 1 |
| Authors | 3b | Describe contributions of protocol authors and identify the guarantor of the review | Yes | 17 |
| Authors | 3c | Describe contributions of the guarantor of the review | Yes | 17 |
| Amendments | 4 | If the protocol represents an amendment of a previously completed or published protocol, identify as such and list changes; otherwise state plan for documenting amendments | Yes | 4 |
| Support | 5a | Indicate sources of financial or other support for the review | Yes | 17 |
| Support | 5b | Provide name for the review funder and/or sponsor | Yes | 17 |
| Support | 5c | Describe roles of funder(s), sponsor(s), and/or institution(s), if any, in developing the protocol | Yes | 17 |
| Introduction | 6 | Describe the rationale for the review in the context of what is already known | Yes | 2–4 |
| Introduction | 7 | Provide an explicit statement of the question(s) the review will address with reference to participants, interventions, comparators, and outcomes | Yes | 5 |
| Methods | 8 | Specify the study characteristics and report characteristics to be used as criteria for eligibility for the review | Yes | 10 |
| Information sources | 9 | Describe all intended information sources, with planned dates of coverage | Yes | 5 |
| Search strategy | 10 | Present draft of search strategy to be used for at least one electronic database | Yes | 6–8 |
| Study records | 11a | Describe the mechanism(s) that will be used to manage records and data throughout the review | Yes | 7 |
| Study records | 11b | State the process that will be used for selecting studies through each phase of the review | Yes | 7–8 |
| Study records | 11c | Describe planned method of extracting data from reports | Yes | 10 |
| Data items | 12 | List and define all variables for which data will be sought | Yes | 10 |
| Outcomes and prioritization | 13 | List and define all outcomes for which data will be sought, including prioritization of main and additional outcomes | Yes | 8–11 |
| Risk of bias in individual studies | 14 | Describe anticipated methods for assessing risk of bias of individual studies | Yes | 12-13 |
| Data synthesis | 15a | Describe criteria under which study data will be quantitatively synthesized | Yes | 10, 13 |
| Data synthesis | 15b | If data are appropriate for quantitative synthesis, describe planned summary measures, methods of handling data, and methods of combining data from studies | Yes | 13 |
| Data synthesis | 15c | Describe any proposed additional analyses | Yes | 13 |
| Data synthesis | 15d | If quantitative synthesis is not appropriate, describe the type of summary planned | Yes | 11, 13 |
| Meta-bias(es) | 16 | Specify any planned assessment of meta-bias(es) | Yes | 13 |
| Confidence in cumulative evidence | 17 | Describe how the strength of the body of evidence will be assessed | No | Not reported |
